# Screening tools to expedite assessment of frailty in people receiving haemodialysis: a diagnostic accuracy study

**DOI:** 10.1186/s12877-021-02356-x

**Published:** 2021-07-02

**Authors:** Tobia Zanotto, Thomas H. Mercer, Marietta L. van der Linden, Pelagia Koufaki

**Affiliations:** 1 Centre for Health, Activity and Rehabilitation Research, School of Health Sciences, Queen Margaret University, Edinburgh, EH21 6UU UK; 2 Present Address: Department of Physical Therapy and Rehabilitation Science, University of Kansas Medical Center, 3901 Rainbow Blvd, Kansas City, KS, 66103 USA

**Keywords:** Frailty, Elderly, frail, Accidental falls, Kidney failure, chronic, Hemodialysis

## Abstract

**Background:**

Frailty is associated with multiple adverse outcomes in stage-5 chronic kidney disease (CKD-5) and upwards of one third of people receiving haemodialysis (HD) are frail. While many frailty screening methods are available in both uremic and non-uremic populations, their implementation in clinical settings is often challenged by time and resource constraints. In this study, we explored the diagnostic accuracy of time-efficient screening tools in people receiving HD.

**Methods:**

A convenience sample of 76 people receiving HD [mean age = 61.1 years (SD = 14), 53.9% male] from three Renal Units were recruited for this cross-sectional study. Frailty was diagnosed by means of the Fried phenotype. Physical performance-based screening tools encompassed handgrip strength, 15-ft gait speed, timed up and go (TUG), and five-repetition sit to stand (STS-5) tests. In addition, participants completed the SF-36 Health Survey, the short-form international physical activity questionnaire and the Tinetti falls efficacy scale (FES) as further frailty-related measures. Outcome measures included the area under the curve (AUC), sensitivity, specificity, positive (PPV) and negative predictive values (NPV). The diagnostic performance of screening tools in assessing fall-risk was also investigated.

**Results:**

Overall, 36.8% of participants were classified as frail. All the examined instruments could significantly discriminate frailty status in the study population. Gait speed [AUC = 0.89 (95%CI: 0.81–0.98), sensitivity = 75%, specificity = 93%] and TUG [AUC = 0.90 (95%CI: 0.80–0.99), sensitivity = 89%, specificity = 85%] exhibited the highest diagnostic accuracy. There was a significant difference in gait speed AUC (20%, *p* = 0.013) between participants aged 65 years or older (*n* = 36) and those under 65 years of age (*n* = 40), with better discriminating performance in the younger sub-group. The Tinetti FES was the only instrument showing good diagnostic accuracy (AUCs≥0.80) for both frailty (sensitivity = 82%, specificity = 79%) and fall-risk (sensitivity = 82%, specificity = 71%) screening.

**Conclusions:**

This cross-sectional study revealed that time- and cost-efficient walking performance measures can accurately be used for frailty-screening purposes in people receiving HD. While self-selected gait speed had an excellent performance in people under 65 years of age, TUG may be a more suitable screening method for elderly patients (≥65 years). The Tinetti FES may be a clinically useful test when physical testing is not achievable.

## Background

Frailty is a biological syndrome of decreased reserve and resistance to stressors, resulting from cumulative declines across multiple physiologic systems, and causing vulnerability to adverse outcomes [1]. Chronic kidney disease (CKD) promotes the activation of multiple pro-ageing pathways, which can lead to an early onset of frailty and increase the risks for morbidity and mortality [2]. Progression to stage-5 chronic kidney disease (CKD-5) is associated with further worsening of physical function and frailty-related outcomes [3]. Several observational studies have consistently concluded that upwards of one third of people receiving haemodialysis (HD) meet diagnostic criteria for frailty [4]. In the context of CKD-5-HD, frailty has been associated with multiple adverse outcomes such as loss of functional independence, falls, hospitalisations, cognitive impairment, vascular access complications, lower chances of receiving a kidney transplant and increased risk of mortality [5–9].

Despite the overwhelming clinical implications of frailty, appropriate screening is still not routinely performed in many HD Units [10]. Several screening tools have been proposed and validated in at-risk populations living with or without CKD [11, 12]. However, the two most common operationalisations of frailty are the Fried phenotype [1] and the deficit accumulation model, as assessed through the Frailty Index [13]. While both approaches have their unique strengths, the Fried phenotype remains the de facto gold standard [14] due to its earlier introduction and greater evidence in terms of predicting negative health outcomes in CKD [15]. Although the Fried phenotype is a relatively expedient assessment, it requires a combination of both physical measures and questionnaires. For this reason, many clinicians still find this procedure time-consuming and potentially unpractical in the context of renal outpatient services [12]. To overcome this implementability issue, several researchers have designed alternative operationalisations of the Fried phenotype by replacing the performance-based measures with subjective (questionnaire-based) assessments in both CKD [16] and non-CKD populations [17, 18]. Although these self-reported definitions of frailty perform well in predicting adverse outcomes [5], they are often less accurate than objective assessments of physical performance in diagnosing frailty in people living with CKD-5 [12, 16]. Therefore, while self-reported measures remain advantageous from a practical standpoint, there is also a need to identify objective measures of physical performance which could be conveniently utilised, as an alternative to the Fried phenotype, in renal outpatient services. In this regard, several “field” performance-based tests such as gait speed, timed up and go, and repeated chair stands are commonly employed to assess physical function in CKD [19] and may represent a viable solution.

Assessing frailty in HD is becoming increasingly important due to the rapid ageing of dialysis populations [20]. Early identification of frailty may be a valuable strategy to improve overall outcomes, while repeated assessments over time can provide useful prognostic information and assist both nephrologists and patients in better understanding the risks and benefits of dialysis continuation in frail individuals [10, 14]. The emerging need to routinely and accurately evaluate frailty is accompanied by the call for identification of screening tools that are less time-intensive (compared to reference standards), easily implementable in HD settings, and predictive of multiple frailty-related outcomes [16]. Therefore, the objective of this study was to explore the diagnostic accuracy of several frailty screening methods, using the Fried phenotype as reference standard, in people receiving HD. As a secondary objective, we examined the diagnostic accuracy of the proposed methods in predicting fall-risk, often a corollary of frailty, within the same population.

## Methods

### Study design and setting

A cross-sectional study design was used to explore the diagnostic accuracy of frailty-related screening tools (e.g. objective and subjective measures of physical function), utilising the Fried phenotype as reference standard, in a convenience sample of people receiving HD. The study was conducted in three Renal Units located in Fife and North Lanarkshire, United Kingdom, between October 2015 and August 2018 (trial registration ID: NCT02392299). All frailty-related and clinical measures were collected during a single assessment, which was performed by a trained researcher on a non-dialysis day (during the midweek interval). The study conformed to the ethical principles for medical research involving human participants, as outlined by the world medical association declaration of Helsinki, and received ethical approval by the Queen Margaret University and West of Scotland NHS Research Ethics Committees (NHS REC reference number: 15/WS/0079).

### Study participants

A convenience sample of prevalent CKD-5 patients receiving HD therapy was recruited for this study. Inclusion criteria were: 1) HD vintage of at least 3 months, 2) good understanding of spoken and written English, and 3) aged 18 years or older. Patients were not considered eligible if they had 1) lower limb amputation without prosthesis, 2) unstable cardiovascular conditions (i.e. clinically severe left ventricular outflow obstruction, suspected or known aneurysm, critical mitral stenosis, critical cerebrovascular stenosis, critical proximal coronary artery stenosis), 3) unstable dialysis and medication treatment, 4) severe cognitive impairment (defined by clinical diagnoses ascertained through medical records, e.g. dementia, Alzheimer’s disease), and 5) pregnancy. People who agreed to take part in the study provided written informed consent prior to participation.

### Data collection procedures

Demographics (i.e. age, gender) and clinical characteristics (i.e. HD vintage, Charlson comorbidity index, number of medications and laboratory values) were extracted from the participants’ medical records. Height, weight and body mass index were measured on the assessment day. Falls were operationally defined according to the Prevention of Falls Network Europe (ProFaNE) recommendations as unexpected events in which the participant comes to rest on the ground, floor, or lower level [21]. We utilised a customised falls questionnaire to prospectively record falls for 12 months. A trained researcher administered this questionnaire to participants once a month, during their dialysis sessions [22]. Participants were classified at-risk of falling if they 1) experienced at least one fall during the prospective follow-up, or 2) reported at least two falls in the previous year [23, 24].

Frailty was operationalised by means of the Fried phenotype [1], which assesses the five canonical components of unintentional weight loss, exhaustion, weakness, slow walking speed and low levels of physical activity. These components were defined as: 1) unintentional weight loss ≥10 lbs. in the previous year (ascertained through medical records), 2) self-reported exhaustion, assessed by means of the SF-36 questionnaire (vitality score <55) [25], 3) low strength, assessed through an isometric handgrip test below an established threshold [1], 4) low gait speed, assessed as time to walk 15 ft above an established threshold [1], and 5) low self-reported levels of physical activity, assessed by means of the short-form international physical activity questionnaire (IPAQ-SF) [26] (total Kcal/week below an established threshold [1]). Participants were classified as frail if they met at least three of these components [1, 27]. Among non-frail participants, individuals who met one or two criteria were classified as pre-frail, while those not meeting any criteria were considered robust.

Participants completed a battery of physical function tests including the handgrip test, 15-ft walking test, three-metre timed up and go (TUG) test, and five-repetitions sit to stand test (STS-5), which were used as frailty screening tools [11, 12]. Maximal isometric handgrip strength was measured, as part of the Fried phenotype, by means of a hydraulic hand dynamometer (Jamar Patterson Medical Ltd., USA) in the seated position with the elbow flexed at 90 degrees and the forearm in the neutral position: participants performed three trials with the dominant arm, interspersed by a one-minute rest, and the average of these was taken for analysis [28]. The 15-ft walking test was used to assess gait speed (m/s) [1]. The beginning and ending of the 15-ft track (4.57 m) were marked with adhesive tape, and a stopwatch was used to record the time. Participants were instructed to walk with their normal walking pace and were allowed to use their assistive walking devices (e.g. cane) if necessary. Two trials interspersed by a 30-s rest were performed and the average was taken for data analysis. For the TUG test, participants were asked to stand up from a chair, walk three metres, turn, walk back to the chair and sit down again, as quickly as possible [29]. This test was executed twice and up to 60 s of rest were allowed between the two measurements. The average time to perform the two trials was calculated for data analysis [30]. Finally, participants performed the STS-5 test as a further measure of lower limb muscle power [31]. The time required to rise from a chair repeatedly five times, as quickly as possible was recorded [32]. One trial rounded to the hundredth of a second was taken for data analysis.

Participants were also administered the SF-36 Health Survey 2.0, the IPAQ-SF and the Tinetti Falls Efficacy Scale (FES) as further frailty-related measures [33–35] by the trained researcher. The SF-36 is a validated tool for the assessment of health-related quality of life in both CKD and non-CKD populations [36]. This survey evaluates eight domains of health: physical functioning, role limitations due to physical health problems, bodily pain, general health, vitality, social functioning, role limitations due to emotional problems, and mental health [37]. Participants completed the SF-36 and their answers to the questions were transformed to create scores (ranging from 0 to 100) for each domain, using appropriate SPSS syntaxes. Physical and mental composite scores were also calculated as per standard procedures [37]. The scores from the physical functioning domain (SF-36 PF) and the physical component summary (SF-36 PCS) were taken for analysis [33]. The IPAQ-SF is a four-item questionnaire asking about frequency and duration of walking activities, moderate- and vigorous-intensity activities, and sedentary behaviour (average daily sitting-time) in the last 7 days [26]. The frequency and duration of these activities were initially entered as ‘days’ and ‘minutes’, which were subsequently converted to MET-minutes/week by using a physical activity compendium, as per standard procedures [38]. Finally, participants were administered the Tinetti FES, a 10-item rating scale assessing perceived levels of confidence in undertaking a range of activities of daily living (ADL) without fear of falling [39]. Participants were asked to rate their confidence from one to 10 for each ADL, with higher scores indicating worse confidence and higher fear of falling.

### Statistical analysis

Statistical analyses were performed with SPSS (Version 26 for Windows, SPSS Inc., Chicago, IL). The Kolmogorov-Smirnov test was used to assess whether data were normally distributed. Demographic and clinical characteristics were summarised as mean ± standard deviation or median and interquartile range based on normal distribution assumptions. Individual missing items were handled with pairwise deletion in the analysis. Differences between frail and non-frail participants were explored by means of Independent t-tests and Mann-Whitney U for continuous variables, as appropriate, or through Chi-square tests/Fisher’s exact test for categorical variables. Receiver operating characteristic (ROC) analysis was used to explore the diagnostic accuracy of screening tools through the area under the curve (AUC). Classifier evaluation metrics included the Gini Index, the KS statistic, and test cut-offs along with their sensitivity/specificity. The positive/negative predictive (PPV/NPV) values and likelihood ratios (LR) were also determined. In a further analysis, we explored the diagnostic accuracy of the screening tools categorised by age (< 65 years old and ≥ 65 years old) and we compared the AUCs in the two age groups. Additional ROC analyses were performed to explore the diagnostic performance of the screening tools, as well as the Fried phenotype, in assessing fall-risk. Statistical limits for interpretation of all analyses were set at an alpha level of 0.05.

## Results

### Study participants

Seventy-six people [mean age = 61.1 years (SD = 14), 53.9% male] undergoing outpatient HD therapy at the Renal Units volunteered to take part in this cross-sectional study. Overall, 28 participants (36.8%) were classified as frail using the Fried phenotype descriptions. The remaining 48 participants (63.2%) were classified as non-frail, with 42 (55.3%) and six (7.9%) meeting the criteria for pre-frailty and robustness respectively. The demographic and clinical characteristics of frail and non-frail participants are summarised in Table 1. Those who were frail had higher age, Charlson comorbidity index, number of prescribed medications, a higher proportion of falls and lower levels of albumin and creatinine compared to their non-frail counterparts (Table 1). Two participants (2.6%) did not provide complete answers to the SF-36 questionnaire and were therefore excluded from the calculation of the PF and PCS subscales. One (1.3%), two (2.6%) and five (6.6%) frail participants were unable to perform the gait speed, TUG and STS-5 tests, respectively.

**Table 1:** Demographic and clinical characteristics of study participants: results are expressed as percentages for categorical variables and mean ± SD or median [IQR] for continuous variables

Variables	Frail (28)	Non-frail (48)	***P***-value
Gender, female, n (%)	11 (39.3)	24 (50)	0.366
Age (years)	66.5 ± 10.5	57.9 ± 14.9	0.009
BMI (kg * m^−2^)	28.6 ± 6.3	29.2 ± 6.4	0.672
Dialysis vintage (days)	449 [881]	497 [891]	0.690
CCI (score)	6 ± 2.1	4.8 ± 2.3	0.032
Diabetes mellitus, n (%)	9 (32.1)	11 (22.9)	0.378
Vascular access type, n (%)			
*Arteriovenous fistula*	15 (53.6)	35 (74.5)	0.063
*Central-venous*	13 (46.4)	12 (25.5)	0.063
Medications (n°)	13.3 ± 4.5	10.9 ± 2.9	0.015
History of falls, n (%)	16 (59.3)	17 (35.4)	0.046
Laboratory values			
*Hb (g/dL)*	11.2 ± 1.1	11.2 ± 1.2	0.924
*CRP (mg/L)*	8 [24]	6 [8]	0.075
*Phosphate (mmol/L)*	1.3 ± 0.5	1.5 ± 0.6	0.084
*PTH (ρmol/L)*	24 [19.4]	19.1 [16.8]	0.177
*Albumin (g/L)*	36.5 [5.8]	38 [4]	0.015
*Adjusted calcium (mmol/L)*	2.4 ± 0.1	2.3 ± 0.1	0.475
*URR (%)*	70.8 ± 7	71.5 ± 5.4	0.635
*Creatinine (μmol/L)*	555 ± 147	680 ± 150	0.001

*Abbreviations*: *SD* Standard deviation, *IQR* Interquartile range, *BMI* Body mass index, *CCI* Charlson comorbidity index, *HD* Haemodialysis, *Hb* Hemoglobin, *CRP* C-reactive protein, *PTH* Parathyroid hormone, *URR* Urea reduction ratio

### Frailty screening tools

**Fig. 1:**
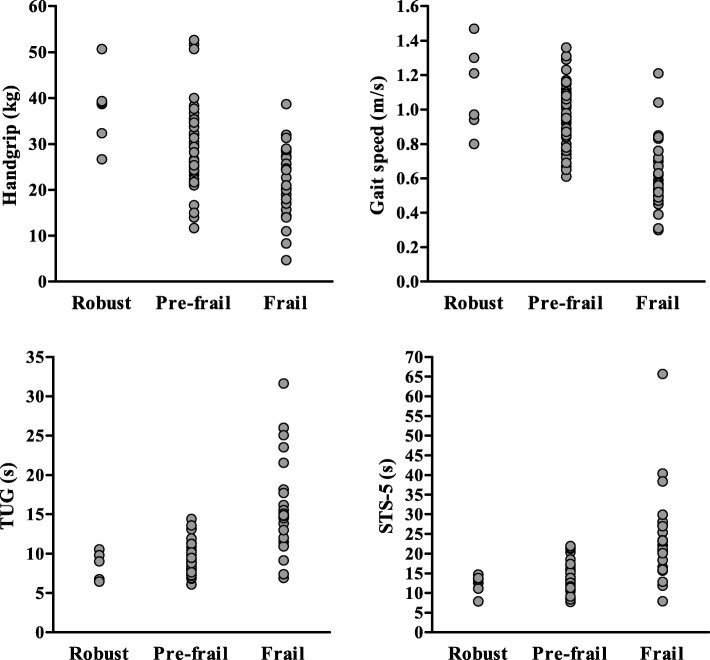
Individual value plots of physical performance-based screening tools in frail and non-frail (robust and prefrail) participants. Legend: TUG: timed up and go test; STS-5: five-seconds sit to stand test

**Fig. 2:**
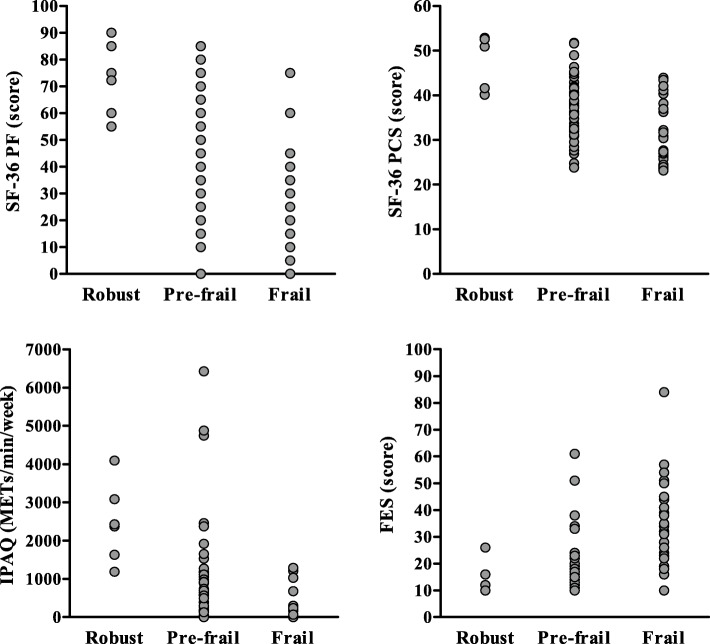
Individual value plots of questionnaire-based screening tools in frail and non-frail (robust and pre-frail) participants. Legend: SF-36 PF: physical function score of SF-36 questionnaire; SF-36 PCS: physical composite scale of SF-36 questionnaire: IPAQ: international physical activity questionnaire (short format); FES: Tinetti falls efficacy scale

Individual value plots of the frailty screening tools data among robust, pre-frail and frail participants are shown in Figs. 1 and 2. The diagnostic accuracies of the screening tools are summarised in Table 2. Overall, gait speed and TUG had the highest AUC values (0.89 [95%CI: 0.81–0.98] and 0.90 [95%CI: 0.80–0.99]), with gait speed having the highest PPV (0.86) and LR+ (10.14), and TUG having the highest NPV (0.93) and lowest LR- (0.13). Among screening tools that were not based on physical performance, the IPAQ-SF and Tinetti FES had the greatest diagnostic accuracy (AUC = 0.84 [95%CI: 0.75–0.94] and AUC = 0.84 [95%CI: 0.74–0.94]). A cut-off value of ≤99 METS/min/week (total physical activity) in the IPAQ-SF had excellent sensitivity (90%) but only fair specificity (71%), while a cut-off value of ≥21 in the Tinetti FES had both good sensitivity (82%) and specificity (79%).

**Table 2:** Diagnostic accuracy of screening tools to expedite assessment of frailty in people receiving haemodialysis

Screening tools	AUC (95% CI)	***P***-value	Gini-I	K-S	Cut-off	Prevalence, n (%)	SENS	SPEC	PPV	NPV	LR+	LR-
Handgrip (Kg)	0.71 (0.59–0.83)	0.001	0.42	0.36	≤ 21.17	18 (23.7)	90%	46%	0.49	0.89	1.67	0.22
Gait speed (m/s)	0.89 (0.81–0.98)	< 0.001	0.78	0.68	≤ 0.85	38 (50.7)	75%	93%	0.86	0.87	10.14	0.27
TUG (s)	0.90 (0.80–0.99)	< 0.001	0.79	0.74	≥ 10.88	30 (40.5)	89%	85%	0.76	0.93	6.06	0.13
STS-5 (s)	0.86 (0.75–0.96)	<0.001	0.71	0.64	≥ 15.65	30 (42.3)	87%	77%	0.64	0.93	3.80	0.17
SF-36 PF (score)	0.78 (0.67–0.89)	< 0.001	0.56	0.49	≤ 42.5	40 (54.1)	64%	85%	0.71	0.80	4.31	0.42
SF-36 PCS (score)	0.76 (0.64–0.88)	< 0.001	0.52	0.47	≤ 32.3	27 (37)	80%	67%	0.59	0.85	2.41	0.29
IPAQ (METs/min/week)	0.84 (0.75–0.94)	< 0.001	0.68	0.61	≤ 99	25 (32.9)	90%	71%	0.64	0.92	3.13	0.15
FES (score)	0.84 (0.74–0.94)	< 0.001	0.68	0.61	≥ 21	33 (43.4)	82%	79%	0.69	0.88	3.95	0.23

*Abbreviations AUC* Area under the curve, *CI* Confidence interval, *Gini-I* Gini Index, *K-S* KS statistic, *SENS* Sensitivity, *SPEC* specificity, *PPV* Positive predictive value, *NPV* Negative predictive value, *LR+* Positive likelihood ratio, *LR*- Negative likelihood ratio, *TUG* Timed up and go test, *STS-5* Five-seconds sit to stand test, *SF-36 PF* Physical function score of SF-36 questionnaire, *SF-36 PCS* Physical composite scale of SF-36 questionnaire, *IPAQ* International physical activity questionnaire (short format), *FES* Tinetti falls efficacy scale

The diagnostic accuracy of the frailty screening tools categorised by age are summarised in Table 3. Overall, the diagnostic accuracies were comparable for most tools in the < 65 years-old (*n* = 40) and ≥ 65 years-old (*n* = 36) sub-groups, with differences in AUCs ranging from 0.02 to 0.11 (*p*-values≥0.277). However, there was a significant difference in AUC for gait speed (0.20 [95%CI: 0.04–0.35], *p* = 0.013), with a better performance of this test in the < 65 years-old sub-group (0.98 [95%CI: 0.96–1.00]) compared to the ≥65 years-old (0.79 [95%CI: 0.64–0.94]). Additionally, while the AUC values of the handgrip were similar in both sub-groups, this test did not significantly discriminate frail from non-frail individuals in those aged under 65 years (0.66 [95%CI: 0.46–0.86]).

**Table 3:** Diagnostic accuracy of frailty screening tools according to age group

Screening tools	AUC (95% CI)	***P***-value	∆AUC (95% CI)	***P***-value
Handgrip				
*< 65 years*	0.66 (0.46–0.86)	0.123	0.08 (−0.34–0.18)	0.564
*≥ 65 years*	0.73 (0.57–0.90)	0.006		
Gait speed				
*< 65 years*	0.98 (0.96–1.00)	< 0.001	0.20 (0.04–0.35)	0.013
*≥ 65 years*	0.79 (0.64–0.94)	< 0.001		
TUG				
*< 65 years*	0.95 (0.87–1.00)	< 0.001	0.08 (−0.08–0.23)	0.350
*≥ 65 years*	0.87 (0.73–1.00)	< 0.001		
STS-5				
*< 65 years*	0.92 (0.82–1.00)	< 0.001	0.11 (−0.09–0.30)	0.277
*≥ 65 years*	0.81 (0.65–0.97)	< 0.001		
SF-36 PF				
*< 65 years*	0.81 (0.67–0.96)	< 0.001	0.03 (−0.18–0.24)	0.786
*≥ 65 years*	0.78 (0.63–0.94)	< 0.001		
SF-36 PCS				
*< 65 years*	0.83 (0.66–0.99)	< 0.001	0.08 (−0.15–0.32)	0.487
*≥ 65 years*	0.74 (0.58–0.91)	0.004		
IPAQ				
*< 65 years*	0.80 (0.63–0.97)	< 0.001	0.06 (−0.27–0.15)	0.549
*≥ 65 years*	0.87 (0.75–0.99)	< 0.001		
FES				
*< 65 years*	0.87 (0.76–0.98)	< 0.001	0.02 (−0.16–0.19)	0.853
*≥ 65 years*	0.85 (0.72–0.99)	< 0.001		

*Abbreviations*: *AUC* Area under the curve, *∆AUC* Difference in area under the curve, *CI* Confidence interval, *TUG* Timed up and go test, *STS-5* Five-seconds sit to stand test, *SF-36 PF* Physical function score of SF-36 questionnaire, *SF-36 PCS* Physical composite scale of SF-36 questionnaire, *IPAQ* International physical activity questionnaire (short format), *FES* Tinetti falls efficacy scale

### Fall-risk screening

Table 4 illustrates the diagnostic accuracy of the examined screening tools for the assessment of fall-risk. Overall, the handgrip, gait speed, TUG, SF-36 and the Tinetti FES could significantly discriminate participants with history of falls from those without falls. The AUC values of handgrip, gait speed, TUG and SF-36 ranged from 0.65 (95%CI: 0.52–0.78) to 0.69 (95%CI: 0.57–0.81), indicating a poor to fair diagnostic value. The Tinetti FES exhibited good diagnostic accuracy (AUC = 0.80 [95%CI: 0.69–0.90], *p* < 0.001), with a cut-off value ≥18 having good sensitivity (82%) and fair specificity (71%). On the other hand, the Fried phenotype did not significantly discriminate fall-risk in the study population (AUC = 0.61 [95%CI: 0.48–0.74], *p* = 0.093).

**Table 4:** Diagnostic accuracy of screening tools to expedite assessment of fall-risk in people receiving haemodialysis

Screening tools	AUC (95% CI)	***P***-value	Gini-I	K-S	Cut-off	Prevalence, n (%)	SENS	SPEC	PPV	NPV	LR+	LR-
Handgrip (Kg)	0.67 (0.54–0.79)	0.009	0.33	0.34	≤ 28.5	46 (60.5)	55%	79%	0.67	0.69	2.58	0.57
Gait speed (m/s)	0.65 (0.52–0.78)	0.021	0.31	0.32	≤ 0.75	27 (36)	79%	53%	0.56	0.77	1.68	0.40
TUG (s)	0.66 (0.53–0.79)	0.015	0.32	0.30	≥ 10.7	31 (41.9)	58%	71%	0.60	0.70	2.03	0.59
STS-5 (s)	0.57 (0.43–0.71)	0.348	0.14	0.25	≥ 20.3	18 (25.4)	39%	86%	0.65	0.68	2.75	0.71
SF-36 PF (score)	0.69 (0.57–0.81)	0.002	0.38	0.33	≤ 27.5	25 (33.8)	81%	52%	0.56	0.79	1.67	0.37
SF-36 PCS (score)	0.66 (0.54–0.79)	0.011	0.33	0.31	≤ 32.9	30 (41.1)	73%	58%	0.57	0.74	1.75	0.46
IPAQ (METs/min/week)	0.54 (0.41–0.67)	0.528	0.08	0.14	≤ 1243	61 (80.2)	26%	88%	0.63	0.60	2.17	0.84
FES (score)	0.80 (0.69–0.90)	< 0.001	0.59	0.53	≥ 18	40 (52.6)	82%	71%	0.69	0.83	2.86	0.25

*Abbreviations*: *AUC* Area under the curve, *CI* Confidence interval, *Gini-I* Gini Index, *K-S* KS statistic, *SENS* Sensitivity, *SPEC* Specificity, *PPV* Positive predictive value, *NPV* Negative predictive value, *LR+* Positive likelihood ratio, *LR-* Negative likelihood ratio, *TUG* Timed up and go test, *STS-5* Five-seconds sit to stand test, *SF-36 PF* Physical function score of SF-36 questionnaire, *SF-36 PCS* Physical composite scale of SF-36 questionnaire, *IPAQ* International physical activity questionnaire (short format), *FES* Tinetti falls efficacy scale

## Discussion

In this study, we explored the diagnostic accuracy of selected screening tools to expedite assessment of frailty in people receiving HD, using the Fried phenotype as the reference standard. Overall, all the examined methods could significantly discriminate frail from non-frail individuals, with gait speed and TUG exhibiting the highest AUC values and elevated PPV/NPV. While gait speed had the highest specificity (93%) and PPV (0.86), TUG had the highest NPV (0.93). As a secondary objective, we explored the diagnostic accuracy of the same methods for fall-risk screening. In this further analysis, the Tinetti FES revealed the highest AUC value.

The prevalence of frailty in the study population was 36.8% which is strongly aligned with findings from a recent meta-analysis on the prevalence of physical frailty in CKD-5 [4]. Therefore, our findings seem to exhibit external validity and may be generalised to the general HD population. Among non-frail participants, only one eighth were classified as robust, while the large majority of patients met at least one of the criteria of the Fried phenotype, which is also in agreement with previous research [40, 41]. Although the mean age of frail participants (66.5 ± 10.5 years) in our sample was considerably lower compared to community-dwelling participants from the Cardiovascular Health Study [1], the prevalence of frailty was about five-fold higher. This observation may indirectly reflect the premature onset of frailty in people living with CKD-5 [2].

Previous diagnostic accuracy studies employed a geriatric assessment [42], a frailty index [43], and the Fried phenotype [12] to evaluate different frailty screening methods in CKD-5 populations. These different choices in terms of reference standards highlight the current lack of consensus on an unequivocal definition of frailty. While a comprehensive geriatric assessment is regarded as the gold standard for the assessment of frailty in clinical practice [44], the Fried phenotype has often been preferred due to its greater expediency and solid evidence base in terms of predicting adverse outcomes. In the study by van Loon et al., [42], 75 and 48% of participants were classified as frail according to a comprehensive geriatric assessment, which was used as reference standard, and to the Fried phenotype, respectively. It is interesting to note how the discrepancy in frailty prevalence emerging from this study was most likely underscored by the different conceptualisations of frailty that were employed. Indeed, the geriatric assessment utilises a multidimensional approach to evaluate multiple components of frailty (e.g. physical and cognitive function, depression, malnutrition, comorbidities etc.) while the Fried phenotype focuses primarily on physical frailty. This important distinction should be kept in mind when interpreting findings from our study. Interestingly, our investigation presents similarities with the work by Nixon et al., [12] in both study design (i.e. Fried phenotype used as the reference standard) and outcomes. In agreement with this study, we found that gait speed had an excellent diagnostic accuracy, with comparable AUC (0.89 vs 0.97), PPV (0.86 vs 0.84) and NPV (0.87 vs 0.96) values. In addition, gait speed performed better than other commonly used performance-based screening tools, such as handgrip strength [12]. Although the sample examined by Nixon et al., [12] predominantly included pre-dialysis patients, our findings seem to corroborate the authors’ conclusion that gait speed can be used to accurately screen for frailty in CKD and, by extension, in the dialysis population.

It is also noteworthy that, while gait speed had excellent overall diagnostic accuracy, there was a significant effect of aging on the observed AUC. Particularly, there was a 20% difference in AUC between age groups, with better performance in those under 65 years of age (Table 3). Since gait speed exhibited lower diagnostic accuracy than TUG in the older group, we plausibly take the view that TUG may be a more suitable screening method in elderly (≥ 65 years-old) patients. Compared to self-selected walking speed, the TUG evaluates more components of physical function such as adequate muscle strength (required to stand up from a chair), ambulation and dynamic balance (required for walking and turning), all of which are negatively affected by aging [45]. It is therefore possible that TUG performance may be more accurate in identifying both the true positives and negatives in the elderly. Interestingly, gait speed, TUG and STS-5 seemed to perform better than the self-reported definition of frailty proposed by Johansen et al., [16], an adaptation of the Fried phenotype based on four (instead of five) criteria. In their study, Johansen et al., [16] reported that such operationalisation of frailty had excellent sensitivity (90%) and NPV (0.93) but only fair specificity (64%) and poor PPV (0.54). From a practical standpoint, the physical performance-based tests examined in our study would offer a more advantageous balance in terms of PPV and NPV while being less time intensive than the self-reported definition. Notably, we observed that a cut-off value of the SF-36 PF ≤ 43 had the best sensitivity-specificity trade-off (Table 2). This contrasted with the cut-off value utilised by Johansen et al., [16] (SF-36 PF < 75), which may explain why their self-report definition of frailty tends to overestimate frailty prevalence [12, 16].

While physical performance tests such as gait speed, TUG and STS-5 could accurately discriminate frailty status, they only exhibited poor to fair accuracy for fall-risk screening (Table 4). Notably, the Fried phenotype did not discriminate fall-risk in our sample, which challenges the suitability of this frailty assessment as a potential gold standard in HD populations. Indeed, some researchers have postulated that some components of the Fried phenotype may not effectively characterise true physiological impairments in people receiving HD. For instance, the unintentional weight loss component may be biased by the fluid shifts at dialysis initiation and by the decreased susceptibility to weight loss in the later stages of CKD-5-HD [41, 46]. This potential confounder could partially explain the lack of diagnostic performance of the Fried phenotype in the study population. Since falls are one of the primary frailty-related outcomes in CKD-5 [10, 47], identifying screening tools that can effectively predict both frailty and fall-risk is paramount in a clinical setting, wherein time and resources constraints often make it unpractical to administer multiple screening tests. In this regard, the Tinetti FES was the only tool showing good diagnostic accuracy for frailty (AUC = 0.84 [95%CI: 0.74–0.94]) and fall-risk screening (AUC = 0.80 [95%CI: 0.69–0.90]) in our study. In addition, this questionnaire performed well as a frailty screener regardless of age, as evidenced by the high AUC values in the ROC analysis categorised by age (Table 3). Therefore, the Tinetti FES may be a valuable tool for clinicians as it combines expediency of frailty screening with useful prognostic information on fall-risk. The Tinetti FES would also have the advantage of not requiring physical testing, which is often a valued feature in clinical settings [16]. Nevertheless, walking-related measures such as gait speed and TUG can also be easily implemented in a clinical setting as they are time/cost effective and require minimal training (of the assessor), resources and patient burden. Additionally, walking speed is an established predictor of mortality in CKD populations [41, 48]. Thus, tests based on walking performance seem to have high overall clinical utility and findings from this investigation strongly suggest that gait speed and TUG are useful frailty screening tools in people receiving HD.

Some strengths and limitations of this study should be carefully examined when interpreting our findings. On the one hand, all frailty-related assessments were conducted by a single researcher on non-dialysis days, which represents a strength in terms of standardisation procedures and potential comparability within the study population. On the other hand, the achieved sample was relatively small (76 participants), which limits the statistical power to detect small sub-group (i.e. age < or ≥ 65 years) effects. In particular, the identification of appropriate cut-offs and their sensitivity/specificity in different age categories would benefit from inclusion of a larger sample. Analogously, due to the modest sample size we could not explore the association between screening tools and mortality. In addition, the convenience sample used in this study could be subjected to selection bias, which may limit the generalisability of findings to the entire CKD-5 population. We should also acknowledge that, due to the physical nature of some of the screening tests employed in the study, we limited our inclusion criteria to participants who had sufficient physical function to perform these tests. The exclusion of more physically impaired patients might have impacted the observed prevalence of frailty as well as the cut-off values identified in ROC analysis. Lastly, the prevalence of frailty may also have been affected by the fact that we replaced the frailty phenotype exhaustion component [1] with the exhaustion criteria proposed by Johansen et al. [25].

## Conclusions

The current study revealed that different time-efficient screening tools involving either physical performance tests or short questionnaires can be used to assess frailty in people receiving HD. Among the examined tools, walking performance measures such as gait speed and TUG exhibited the highest diagnostic accuracy using the Fried phenotype as the reference standard. While gait speed had an excellent diagnostic performance in people under 65 years of age, the TUG may be a more appropriate screening method for elderly patients (≥ 65 years-old). Importantly, the Tinetti FES was the only measure showing good diagnostic accuracy for both frailty and fall-risk screening. The instruments examined in this study could be used to evaluate whether patients may benefit from a comprehensive geriatric assessment. In this regard, further research would be required to explore the diagnostic accuracy of walking performance measures by utilising a geriatric assessment as the reference standard. Ultimately, multiple independent studies may be needed to fathom which screening tools should be incorporated into clinical practice for routine frailty-screening in the dialysis unit.

## Data Availability

The datasets used and analysed during the current study are available from the corresponding author on reasonable request.

## Abbreviations

ADL: Activities of daily living
AUC: Area under the curve
CKD: Chronic kidney disease
CKD-5: stage-5 chronic kidney disease
FES: Falls efficacy scale
HD: Haemodialysis
IPAQ-SF: Short-form international physical activity questionnaire
LR-: Negative likelihood ratio
LR +: Positive likelihood ratio
NPV: Negative predictive value
PPV: Positive predictive value
ROC: Receiver operating characteristic
SF-36 PCS: Physical component summary of SF-36 Health Survey
SF-36 PF: Physical functioning domain of SF-36 Health Survey
STS-5: Five-repetition sit to stand test
TUG: Timed up and go test
